# Development of an Enzyme-Linked Immunosorbent Assay and Gold-Labelled Immunochromatographic Strip Assay for the Detection of Ancient Wool

**DOI:** 10.1155/2018/2641624

**Published:** 2018-06-05

**Authors:** Bing Wang, Jincui Gu, Boyi Chen, Chengfeng Xu, Hailing Zheng, Zhiqin Peng, Yang Zhou, Zhiwen Hu

**Affiliations:** ^1^Key Laboratory of Advanced Textile Materials and Manufacturing Technology, Ministry of Education, Zhejiang Sci-Tech University, Hangzhou 310018, China; ^2^Key Scientific Research Base of Textile Conservation, State Administration for Cultural Heritage, China National Silk Museum, Hangzhou 310002, China; ^3^Institute of Textile Conservation, Zhejiang Sci-Tech University, Hangzhou 310018, China

## Abstract

The identification of ancient wool is of great importance in archaeology. Despite lots of meaningful information can be achieved by conventional detection methods, that is, light and electron microscopy, spectroscopy, and chromatography, the efficacy is likely to be limited in the detection of ancient samples with contamination or severe degradation. In this work, an immunoassay was proposed and performed for the identification of ancient wool. First, a specific antibody, which has the benefits of low cost, easy operation, and extensive applicability, was developed directly through immunizing rabbits with complete antigen (keratin). Then, an enzyme-linked immunosorbent assay (ELISA) and a colloidal gold-labelled immunochromatographic strip (ICS) were developed to qualitatively identify the corresponding protein in ancient wool samples unearthed from Kazakhstan and China. The anti-keratin antibody exhibited high sensitivity and specificity for the identification of modern and ancient wool. The limit of detection (LOD) of the ELISA method was 10 ng/mL, and no cross-reactions with other interfering antigens have been noted. It is concluded that the immunoassays are reliable methods for the identification of ancient wool.

## 1. Introduction

Ancient textiles are an important component of human civilization heritage. Since the Stone Age, in order to adapt to climate change, the ancients had begun to use natural resources as textile materials. Ancient textiles mainly included cotton fibre, hemp fibre, silk, and wool. The earliest evidence for the wool usage in the eastern Iran and in the northern Caucasus dates back to the 4th millennium BC [1]. The importance of wool as a major source of textile and economic trade in Eurasia has long been established [2]. Ancient wool was unearthed from time to time in the graves, tombs, or other places in many countries along the Silk Road [2–4]. The state of preservation mainly depends on the burial time and burial environment of the ancient wool. Under normal circumstances, ancient wool samples recovered from excavations, although fragmentary and fragile, are valuable finds for the study of historical textile production, its trade, and the development of sheep breeding [5].

Wool is a natural composite material consisting of keratin and keratin-associated proteins as key molecular components [6, 7]. The keratin macromolecular structure is the alignment of amino acid residues along its chain. The sequence of amino acids defines the possibility of intermolecular links and the access of amino acids to the chemical reaction [8–10]. However, ancient wool buried in different soil contexts has an intrinsic chemical complexity and the tendency to easily degrade over long periods of time [3, 11]. This is because wool is easily affected by many factors, such as high temperature, oxidation, soil microbes, and radiation, resulting in the degradation of macromolecular chains [12]. Therefore, well-preserved woollen fabrics are rarely found in archaeological contexts, except for environments with special conditions, such as low temperature, anoxia, or extreme dryness. Until recently, species identification and detailed characterization of poorly preserved ancient wool have remained challenging issues in archaeology.

During the past several decades, there were a number of methods available for the identification of archaeological wools, such as scanning electron microscopy [1, 13], Fourier transform infrared spectroscopy [8, 14–16], nuclear magnetic resonance [17], tandem mass spectrometry [18], and gas chromatography-mass spectrometry [19]. However, archaeological wools have usually degraded into short fibres or even peptides, leaving microtraces in the soil. In addition, external contamination and compositional complexity make the identification of ancient samples highly challenging and sometimes highly uncertain. Thus, it is still difficult for archaeologists to extract enough useful information from ancient wool samples.

Immunological techniques have the potential to become a powerful analytical tool in archaeology [20, 21]. These methods offer several advantages over traditional methods used for protein analysis, including low costs, speed, increased sensitivity, and increased specificity [22, 23]. In recent years, immunological techniques have attracted increased attention from professionals involved in the research of cultural heritage [24–27]. All of these studies have demonstrated that immunoassays have the potential to identify and localize the proteins in archaeological materials rapidly and effectively. However, most of the antibodies used in these immunoassays are commercially available, and the need for tailored antibodies for targeting a specific protein with high sensitivity and specificity is highly desirable. Thus, the preparation of a tailored antibody for the detection of ancient wool is compelling, yet challenging.

In our previous research, several tailored specific antibodies were designed for the immunodetection of ancient silks [28, 29], leathers [30], and proteinaceous binders [31]. Moreover, gold-based and lanthanide-labelled immunochromatographic strip assays were developed for the on-site identification of ancient silks [32, 33]. Both the immunosensors showed high sensitivity and specificity, providing a new protocol for identifying archeological proteinaceous materials.

Herein, an immunoassay is proposed for the microtrace detection of ancient wool. First, an anti-keratin antibody was prepared by immunizing rabbits with complete antigen, and the sensitivity and specificity of the antibody were determined to verify its validity for the identification of wool samples. Then, the antibody-based immunoassays, an enzyme-linked immunosorbent assay (ELISA), and a colloidal gold-labelled immunochromatographic strip (ICS) were established and used to qualitatively identify samples from different time periods. ELISA is a powerful analytical laboratory tool for archaeology and conservation, while ICS is especially suitable for the identification of poorly preserved ancient samples in archaeological sites. Consequently, the two methods are particularly useful when used in tandem.

## 2. Materials and Methods

### 2.1. Archaeological Samples

**Figure 1:**
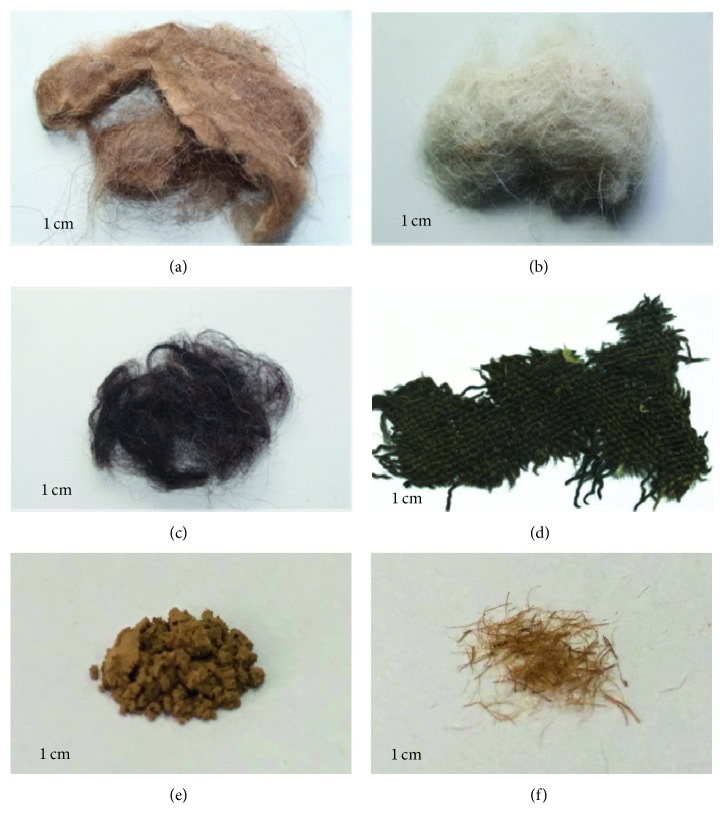
Digital images of ancient samples unearthed in Kazakhstan: (a) camel hair, unearthed in the western Kazakhstan in 1970s; (b) white wool and (c) black wool, unearthed from the Shymkent region in the South Kazakhstan; (d) wool fabrics, unearthed from the Almaty region in Kazakhstan; (e) wool soil sample and (f) wool fibre, unearthed from the Small River Cemetery, Xinjiang, China. These images were taken by a Canon EOS700D digital camera in micromode.

Several precious archaeological samples excavated from Kazakhstan and China were selected for immunological identification. Samples were provided by China National Silk Museum. The original condition of samples is shown in Figure 1. Sample I (Figure 1(a)), which was unearthed from the western Kazakhstan in the 1970s, was camel hair. Sample II (Figure 1(b)) and sample III (Figure 1(c)) were unearthed from the Shymkent region in the South Kazakhstan during the 2000s and 2010s. Sample IV (Figure 1(d)) was unearthed from the Almaty region in Kazakhstan during the 2000s and 2010s. Sample V (Figure 1(e)) and sample VI (Figure 1(f)) were unearthed from the Small River Cemetery (2000∼1450 BC), located in the southwest desert of the Lop Nor Area in Xinjiang, China.

### 2.2. Reagents and Chemicals

Goat anti-rabbit IgG (*H* + *L*) HRP-conjugated secondary antibody (100 *µ*g at 1 mg/mL) and a TMB colour system were purchased from Hangzhou Hua'an Bio-Technology Co., Ltd. Bovine serum albumin (BSA), human serum albumin (HSA), chicken ovalbumin, and collagen were purchased from Sigma-Aldrich. Natural wool was obtained from Hangzhou Fusigongmao Co., Ltd. Natural silk was obtained from Zhejiang Misai Silk Co., Ltd. Hemp fibre and cotton fibre were obtained from Hangzhou Fusi Industry and Trade Co., Ltd. NaOH, H_2_O_2_ (30%), KCl, KH_2_PO_4_, NaCl, Na_2_CO_3_, CaCl_2_, and Na_2_HPO_4_ were purchased from Hangzhou High Crystal Special Chemicals Co., Ltd. Standard soil samples were obtained from Beijing Century Aoke Biotechnology Co., Ltd.

A carbonate bicarbonate buffer solution (pH 9.6) was used as a diluent for ELISA antigens. Phosphate-buffered saline (PBS, pH 7.4) was used for wash steps. 1% BSA in PBS (pH 7.4) was used to block the unbound sites of antigens. All other reagents were of analytical grade and used as received. The water used in all experiments was purified by a Milli-Q water system.

### 2.3. Extraction and Characterization of Antigens

Keratin was extracted from natural wool by an alkali hydrolysis method. Briefly, an alkaline solution was prepared by mixing 4% (w/w) NaOH with an equal volume of 0.6% (w/w) H_2_O_2_ in advance. Then, natural wool was shredded and immersed in the mixed solution at a bath ratio of 1 : 50 at 50°C. After stirring at 200 rpm for 4 h, the wool was completely dissolved, and a clear liquid was obtained. Next, the resultant keratin solution was dialyzed for 72 h with a molecular weight cutoff of 3500 Da and freeze-dried. Then, the obtained keratin was analyzed by SDS-PAGE and FTIR.

All possible interfering antigens, that is, silk fibroin, sericin, cotton fibre, and hemp fibre, were extracted using the previously reported procedures [28].

### 2.4. Preparation of Anti-Keratin Primary Antibody

An anti-keratin antibody was produced by animal immunization as follows. The immunogen (keratin) was first mixed with an equal volume of complete Freund's adjuvant to form an emulsion. For the initial immunization, New Zealand white rabbits (14–16 weeks old) were subcutaneously injected multiple times into their thighs with 100 *µ*L of the above mixture. Then, incomplete Freund's adjuvant was substituted for the complete Freund's adjuvant as an emulsifier for subsequent boosters every 2 weeks. The titre of the antiserum was measured by indirect ELISA ten days after each immunization. Antiserum was collected 6 weeks after the initial immunization and purified by affinity chromatography. The resultant anti-keratin antibody was stored (3.15 mg/mL) at −20°C before use.

All animal experiments were carried out in accordance with the national standard “Laboratory Animal-Requirements of Environment and Housing Facilities” (GB 14925–2001) and the guidelines issued by the Ethical Committee of Zhejiang Sci-Tec University.

### 2.5. Indirect ELISA Test

**Figure 2:**
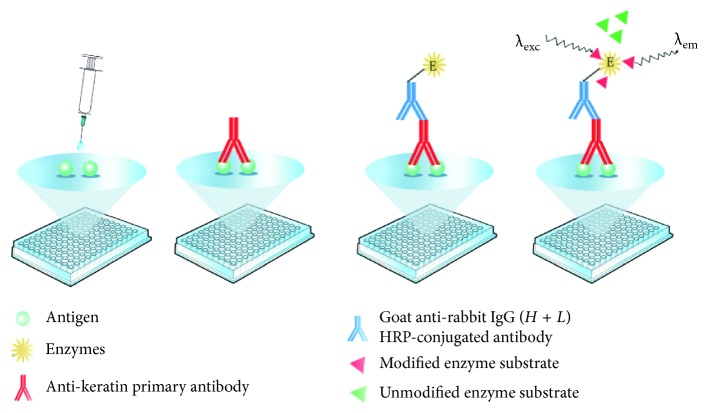
Schematic diagram of the indirect ELISA used for the microtrace detection of ancient wool.

ELISA tests are performed in an indirect format as shown in Figure 2. Samples were first dissolved in a carbonate bicarbonate buffer solution (pH 9.6) and diluted to 10 *μ*g/mL. Then, 100 *μ*L of the sample solution was added to each well of the 96-well microplate and incubated at 4°C overnight. After the solution was removed, the wells were washed 3 times with PBS (7.4). Next, 100 *μ*L of the blocking solution was added to each well and incubated at 37°C for 1 h to occupy the unbound sites, followed by 3 washings with PBS. Then, 100 *μ*L of the anti-keratin antibody was added to the wells and incubated at 37°C for 1 h, followed by washing. Next, 100 *μ*L of secondary antibody (goat anti-rabbit IgG (*H* + *L*) HRP-conjugated) was added, followed by incubation at 37 °C for 1 h. After washing with PBS 3 times, 100 *μ*L of 3, 3′, 5, 5′-tetramethylbenzidine (TMB) was added and incubated in darkness for 10 min. Finally, 100 *μ*L of 1 mol/L H_2_SO_4_ (stopping solution) was added to terminate the colour reaction, and the optical density (OD) of each sample at *λ* = 450 nm was measured using a microplate reader (Model 550, Bio Rad).

Optimal antibody and antigen dilutions corresponding to the best specificity and sensitivity of the ELISA method were obtained by panel titrations. To get the best dilution ratios of antibody and antigen, detailed operation steps were set as follows. The keratin powders were dissolved in a carbonate bicarbonate buffer (pH 9.6) solution that was formulated at concentrations of 1,000 *μ*g/mL, 100 *μ*g/mL, 10 *μ*g/mL, and 1 *μ*g/mL. Then, the anti-keratin primary antibody was diluted by 1% BSA solution at dilution ratios of 1 : 200, 1 : 500, 1 : 1000, 1 : 3,000, 1 : 5,000, and 1 : 10,000, respectively. Finally, the mean OD values of different keratin concentrations with different dilutions of primary antibody were tested by a microplate reader (Model 550, Bio Rad). The optical density of the samples at 450 nm was abbreviated as OD_450_ _nm_.

A series of controls were set up to ensure the specificity of the indirect ELISA test. PBS replaced sample solutions as a coating antigen for the negative control, and only experiments that presented negative results were considered to be reliable. Keratin was employed as a positive control. For the blank control, the anti-keratin primary antibody was replaced with PBS to ensure that the HRP-conjugated secondary antibody did not react with the coating antigens.

### 2.6. Preparation of the Immunochromatographic Strip

The detecting principle of the immunochromatographic strip is based on competitive immunoreactions. There are four parts of the immunochromatographic strip: a sample pad, a colloidal gold pad, a nitrocellulose membrane, and an absorbent pad. The colloidal gold-labelled antibody was sprayed onto a polyester fibre pad by a metal spraying machine. Next, wool keratin (1.2 mg/mL) was sprayed onto a nitrocellulose membrane, as the test line, at 1 *µ*g/cm, while the goat anti-rabbit IgG (*H* + *L*) HRP-conjugated secondary antibody (1.5 mg/mL) was sprayed as the control line. Both the polyester fibre pad and the nitrocellulose membrane were dried at 37°C for 2 h. Finally, all four parts were fitted together, cut into 3.5 mm wide strips, and put into a PVC case. When sample solutions were added dropwise onto the sample pad, they flowed chromatographically along the nitrocellulose strip and gave positive or negative reactions.

### 2.7. Pretreatment of Archaeological Samples for ICS Detection

The extraction of the ancient samples can be summarized as follows. Two milligrams of each sample was added into 100 mL of extracted solutions (including 2% (w/w) sodium hydroxide and 0.3% (w/w) hydrogen peroxide) and incubated for 1 h. Then, the supernatant of each extracted solution was collected and transferred individually into centrifuge tubes for ICS detection.

### 2.8. Statistical Analysis

Five parallel samples were used for each sample and control and were run simultaneously. All values are expressed as the mean ± standard deviation.

## 3. Results and Discussion

### 3.1. Characterization of Keratin

**Figure 3:**
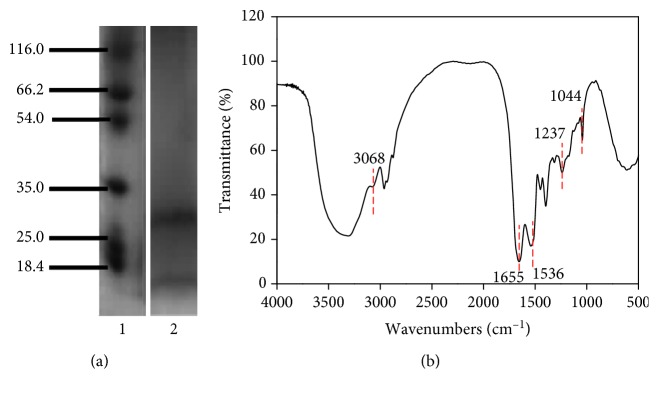
(a) SDS-PAGE analysis of keratin extracted from wool: lane 1: molecular weight marker; lane 2: keratin. (b) FTIR spectrum of keratin.

The molecular weight distribution of the resultant keratin was determined by SDS-PAGE. In terms of the molar mass and sulphur content, four fractions, that is, the low sulphur fraction (LSF, Mw: 45–60 kDa), the high sulphur fraction (HSF, Mw: 14–28 kDa), ultrahigh sulphur fraction (USF, Mw: 37 kDa), and the high Gly/Tyr fraction (HGT, Mw: 9–13 kDa) can be extracted from wool keratin. As shown in Figure 3(a), two bands that match the distribution characteristics of the typical keratin bands, including the HSF chain at approximately 28 kDa and the HGT at approximately 12 kDa, were observed, indicating that the resultant keratins were mainly the HSF and HGT fractions. The chemical structure of the resultant keratin was also characterized via FTIR. As shown in Figure 3(b), the sample spectra show the characteristic peaks of amide bonds, specifically a sharp peak at 1655 cm^−1^ (amide I band), a peak at 1536 cm^−1^ (amide II band), and a peak at 1237 cm^−1^ (amide III band). In particular, a peak at 1044 cm^−1^ appears in the spectrum, which is assigned to the characteristic peak of cysteine sulfenate produced by oxidation of disulfide bond.

### 3.2. The Optimal Dilution Multiple of Antibody and Antigen

**Figure 4:**
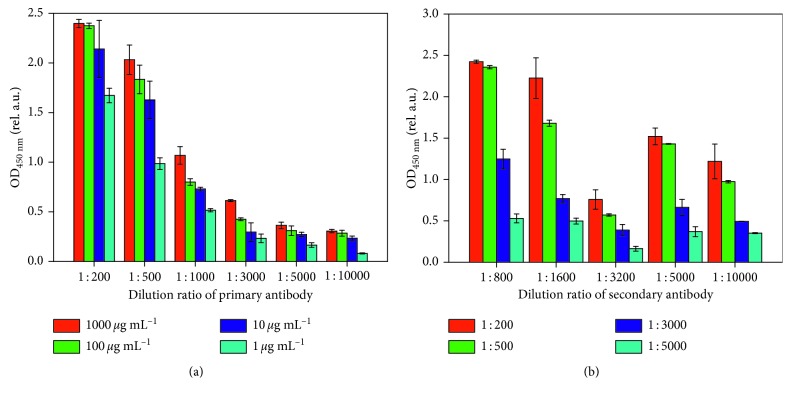
ELISA titration results in different conditions. (a) The dilution ratio of anti-keratin primary antibody is 1 : 200, 1 : 500, 1 : 1,000, 1 : 3,000, 1 : 5,000, and 1 : 10,000, while the concentration of keratin is 1,000 *μ*g/mL, 100 *μ*g/mL, 10 *μ*g/mL, and 1 *μ*g/mL, respectively. (b) The secondary antibody dilution ratio is 1 : 800, 1 : 1,600, 1 : 3,200, 1 : 5,000, and 1 : 10,000, while the anti-keratin primary antibody dilution ratio is 1 : 200, 1 : 500, 1 : 3,000, and 1 : 5,000, respectively. Each column of the figure represents the mean ± SD (standard deviation) of *n*=5 assays.

Investigation of the ELISA procedure for recognition of ancient wool began with an antibody panel titration of standard solutions of keratin. As shown in Figure 4(a), with increasing dilution of the primary antibody, the OD_450_ _nm_ values decreased. Obviously, when the dilution ratio was 1 : 200 or 1 : 500, the OD_450_ _nm_ values were larger at various concentrations of keratin. In addition, if the OD_450_ _nm_ values of keratin ranged from 1.5 relative arbitrary units to 2.0 relative arbitrary units, subsequent ELISA tests would show much higher accuracy. Considering the cost of the primary antibody, the dilution ratio of the anti-keratin primary antibody was set at 1 : 500, and the chosen concentration of keratin was 100 *μ*g/mL for the following experiments. To achieve the optimized dilution of the secondary antibody, the concentration of keratin was set at 100 *μ*g/mL, and the anti-keratin primary antibody and the HRP-conjugated goat anti-rabbit IgG (*H* + *L*) secondary antibody were diluted to various dilution ratios with 1% BSA solution. As shown in Figure 4(b), the OD_450_ _nm_ values decreased with the increase of the dilution ratios of primary antibody and secondary antibody. Paradoxically, under the dilution ratio of 1 : 3,200 for the secondary antibody, the OD_450_ _nm_ values were much smaller than the others. After the exclusion of experimental error, it is speculated that it may result from decreasing immune response of secondary antibody, though further research is needed. When the dilution ratio of secondary antibody was 1 : 1600 or 1 : 5,000, the OD_450_ _nm_ values ranged from 1.5 relative arbitrary units to 2.0 relative arbitrary units. Considering the antibody titre and production costs, the dilution ratio of the secondary antibody was set as 1 : 5,000. To summarize, the optimal dilution factors of the anti-keratin primary antibody and the secondary antibody were 1 : 500 and 1 : 5,000, respectively.

### 3.3. The Specificity of Primary Antibody

**Figure 5:**
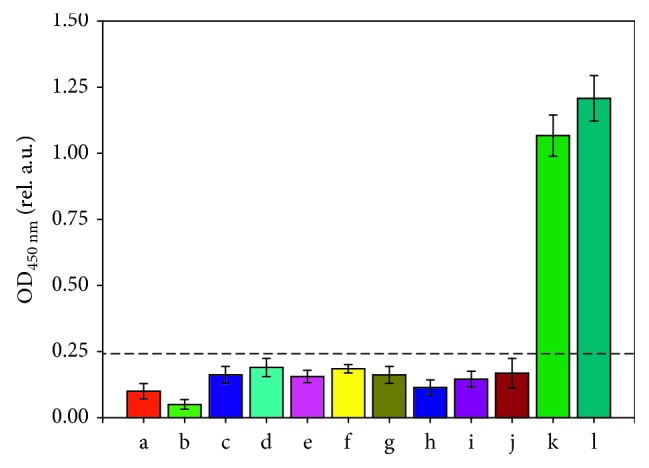
ELISA results for interfering antigens to test the specificity of anti-keratin primary antibody: (a) PBS; (b) negative control; (c) BSA; (d) HSA; (e) ovalbumin; (f) collagen; (g) sericin; (h) fibroin; (i) cotton extraction; (j) hemp extraction; (k) wool extraction; (l) keratin. Each column of the figure represents the mean ± SD (standard deviation) of *n*=5 assays. The dashed line shows the test criterion (the mean OD_PBS_ plus three times the corresponding standard deviation). If OD_450 nm_ is above the dashed line, the result is positive, and vice versa.

Next, the specificity of the anti-keratin primary antibody was further investigated by checking cross-reactivity with other interfering antigens. The limit of detection (LOD) was set as the mean OD_450 nm_ value of negative control plus three times the corresponding standard deviation. As shown in Figure 5, a series of interfering antigens, including BSA, HSA, ovalbumin, collagen, sericin, fibroin, cotton fibre extraction, and hemp fibre extraction (100 *μ*g/mL), were tested. All of the above samples showed clearly negative results, while modern wool extraction and keratin showed positive results. These results indicated that the anti-keratin primary antibody is an appropriate choice for detecting ancient wool due to its high specificity.

### 3.4. The Effectiveness and Sensitivity of Primary Antibody

**Figure 6:**
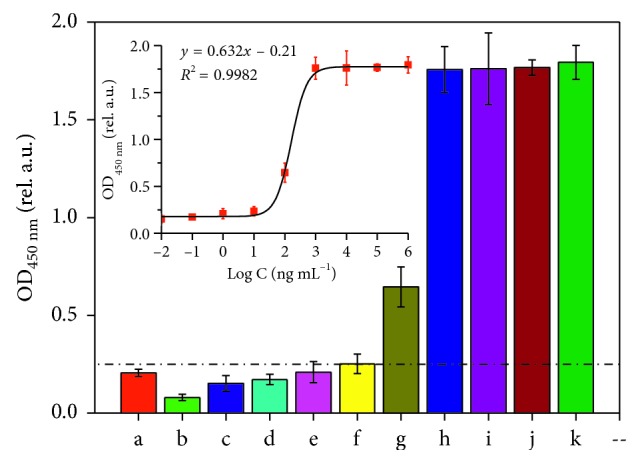
The limit of detection (LOD) of the ELISA test for keratin: (a) PBS; (b) negative control; (c–k) keratin with different concentrations of 10^−2^, 10^−1^, 1, 10, 10^2^, 10^3^, 10^4^, 10^5^, and 10^6^ ng/mL.

An indirect ELISA was then employed to determine the LOD of the primary antibody for keratin. The results are presented in Figure 6. Keratin was diluted to 10^−2^, 10^−1^, 10^0^, 10^1^, 10^2^, 10^3^, 10^4^, 10^5^, and 10^6^ ng/mL with a carbonate bicarbonate buffer (pH 9.6). The results clearly demonstrate that keratin can be detected using the ELISA method when its concentration is above 10 ng/mL and cannot be detected below 10 ng/mL. This finding suggests that the LOD of the primary antibody is 10 ng/mL. Meanwhile, the standard curve, which sets the logarithm of keratin concentration as the abscissa and the OD_450_ _nm_ value as the ordinate, is also shown in Figure 6. The relationship between the logarithm of keratin concentration and the OD_450_ _nm_ value matches the S-type curve model. In particular, the logarithm of keratin concentration and the OD value showed a linear relationship when concentrations of keratin ranged from 10^1^ to 10^3^ ng/mL (OD values ranged from 0.435 to 1.758). Additionally, the linear regression equation was *y* = 0.632*x* − 0.21, *R*^2^ = 0.973. Within this range, the samples could be tested quantitatively by indirect ELISA. These findings proved the effectiveness and high sensitivity of the anti-keratin primary antibody.

### 3.5. Immunodetection of Archaeological Samples with Indirect ELISA Method

**Figure 7:**
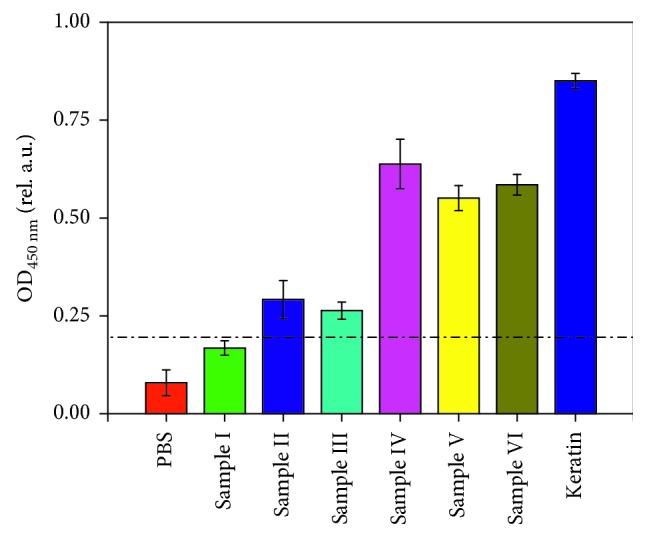
ELISA results for ancient samples.

As the ELISA protocol had been optimized for the identification of keratin, it was employed to detect keratin in archaeological samples. To ensure the reliability of the ELISA tests, PBS was used as a negative control, and keratin served as a positive control. Archaeological samples were extracted using a similar procedure to keratin and were then coated using a carbonate bicarbonate buffer (pH 9.6) solution. As shown in Figure 7, the negative control and sample I showed negative results, while all other ancient samples and positive control (keratin, 100 *μ*g/mL) showed positive results. Because sample I is from camel hair, it could not be detected with this tailored primary antibody. Overall, the ELISA method correctly identified the existence of keratin in ancient wool and wool fabrics, even though they were derived from different species of sheep. The results also proved the effectiveness and specificity of the anti-keratin primary antibody in the ELISA test.

### 3.6. The Limit of Detection (LOD) of Ancient Wool Samples by Indirect ELISA

**Figure 8:**
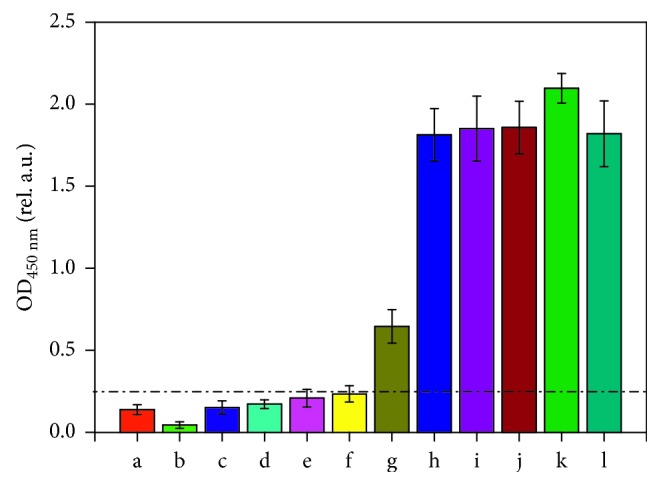
The limit of detection (LOD) of the ELISA test for ancient wool samples: (a) PBS control; (b) negative control; (c–k) ancient wool samples with different concentrations of 10^−2^, 10^−1^, 1, 10, 10^2^, 10^3^, 10^4^, 10^5^, and 10^6^ ng/mL; (l) positive control.

To confirm the ELISA results and decrease the risk of false positives, microtrace detection of ancient wool was carried out. An ancient wool sample was chosen and diluted to 10^−2^, 10^−1^, 10^0^, 10^1^, 10^2^, 10^3^, 10^4^, 10^5^, and 10^6^ ng/mL with carbonate bicarbonate buffer (pH 9.6). The indirect ELISA protocol was then employed to test the LOD for the ancient wool samples. The results are presented in Figure 8. It demonstrates that the LOD of the ancient wool sample was approximately 10 ng/mL. Therefore, if the concentrations of the ancient wool samples were higher than 10 ng/mL, they could be detected directly with the ELISA method. The LOD was the same as that of the modern samples. Although ancient wool samples unearthed from Kazakhstan have been buried for a long time and not only the composition but also the colour has undergone great changes and deterioration, the ELISA method still showed high sensitivity. In fact, as long as the targeted site (epitope) of the antigen remained intact, the anti-keratin primary antibody was able to identify the corresponding protein in ancient wool. Therefore, the deterioration of samples did not have a noticeable impact on the ELISA test.

### 3.7. Immunodetection of Archaeological Samples with ICS Method

**Figure 9:**
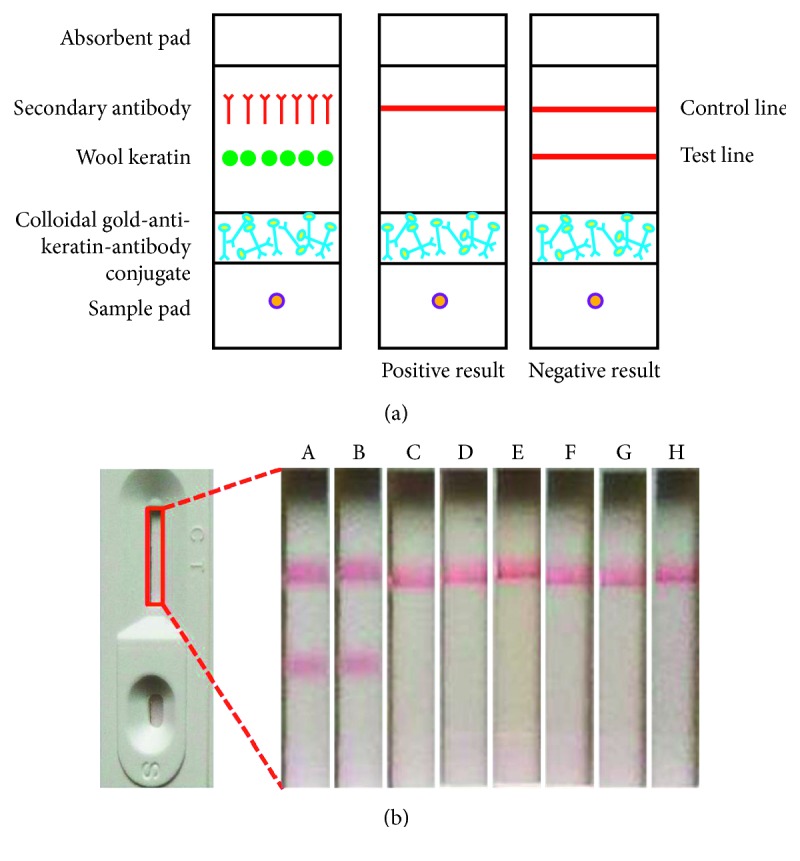
(a) Schematic diagram of the colloidal gold-based immunochromatographic assay; (b) ICS detection results for negative control, positive control, and ancient samples: (A) PBS, (B) sample I, (C) sample II, (D) sample III, (E) sample IV, (F) sample (V) (g) sample VI, and (H) keratin.

The principle of ICS is based on competitive immunoreactions. Wool keratin and HRP-conjugated goat anti-rabbit IgG (*H* + *L*) (secondary antibody) were immobilized on the test line and control line, respectively (Figure 9(a)). As the sample solutions flow chromatographically along the nitrocellulose strip, they will pass the conjugate pad, test line, and control line successively. If both the control line and test line become red, it means that the wool keratin is below the LOD (negative result); if only the control line becomes red, this indicates that the wool keratin is above the LOD (positive result) [32, 33]. As shown in Figure 9(b), six archaeological samples were analyzed using the ICS method, while PBS and keratin were used as negative and positive controls, respectively. For PBS and sample I (camel hair), both the control line and the test line exhibited red colour. In contrast, no red band appeared in the test line for other ancient samples and keratin. The results clearly indicated that the ICS strip could identify wool from other types of archaeological samples. These results were consistent with the ELISA results, confirming the high reliability and practicability of immunoassay.

## 4. Conclusions

This study focused on the microtrace detection of ancient wool with immunological techniques. A specific antibody was developed directly through immunizing rabbits with complete antigen (keratin). Then, antibody-based immunoassays, ELISA, and ICS, were established and conducted to qualitatively identify the corresponding protein in ancient wool. Optimal antibody dilutions corresponding to the best specificity and sensitivity of the ELISA test were obtained by panel titrations. These immunological methods correctly identified the existence of keratin in ancient wool and wool fabrics, even though the samples were from different species of sheep and had been buried underground for a long period. The LOD of both keratin and ancient wool was 10 ng/mL, and no cross-reactions with other possible interference antigens have been noted. Considering the practicality and flexibility of ELISA and ICS, immunological techniques have the potential to become a particularly useful analytical tool for archaeological proteinaceous materials.

## Data Availability

The data used to support the findings of this study are included within the article.
